# Age-related increase of mitochondrial content in human memory CD4+ T cells contributes to ROS-mediated increased expression of proinflammatory cytokines

**DOI:** 10.3389/fimmu.2022.911050

**Published:** 2022-07-22

**Authors:** Yuling Chen, Yuanchun Ye, Pierre-Louis Krauß, Pelle Löwe, Moritz Pfeiffenberger, Alexandra Damerau, Lisa Ehlers, Thomas Buttgereit, Paula Hoff, Frank Buttgereit, Timo Gaber

**Affiliations:** ^1^ Department of Rheumatology and Clinical Immunology, Charité – Universitätsmedizin Berlin, Corporate Member of Freie Universität Berlin, Humboldt-Universität zu Berlin, Berlin, Germany; ^2^ German Rheumatism Research Centre (DRFZ) Berlin, A Leibniz Institute, Berlin, Germany; ^3^ Department of Gastroenterology, Fujian Medical University Affiliated First Quanzhou Hospital, Quanzhou, China; ^4^ Department of Hematology, Oncology and Tumor Immunology, Charité – Universitätsmedizin Berlin, Corporate Member of Freie Universität Berlin, Humboldt-Universität zu Berlin, Berlin, Germany; ^5^ Department of Dermatology, Venerology, and Allergology, Charité – Universitätsmedizin Berlin, Corporate Member of Freie Universität Berlin, Humboldt-Universität zu Berlin, Berlin, Germany; ^6^ Rheumatologie, Endokrinologikum Berlin, Berlin, Germany

**Keywords:** metabolism, aging, memory Th cells, proliferation, cytokines, ROS

## Abstract

Cellular metabolism modulates effector functions in human CD4^+^ T (Th) cells by providing energy and building blocks. Conversely, cellular metabolic responses are modulated by various influences, e.g., age. Thus, we hypothesized that metabolic reprogramming in human Th cells during aging modulates effector functions and contributes to “inflammaging”, an aging-related, chronic, sterile, low-grade inflammatory state characterized by specific proinflammatory cytokines. Analyzing the metabolic response of human naive and memory Th cells from young and aged individuals, we observed that memory Th cells exhibit higher glycolytic and mitochondrial fluxes than naive Th cells. In contrast, the metabolism of the latter was not affected by donor age. Memory Th cells from aged donors showed a higher respiratory capacity, mitochondrial content, and intracellular ROS production than those from young donors without altering glucose uptake and cellular ATP levels, which finally resulted in higher secreted amounts of proinflammatory cytokines, e.g., IFN-γ, IP-10 from memory Th cells taken from aged donors after TCR-stimulation which were sensitive to ROS inhibition. These findings suggest that metabolic reprogramming in human memory Th cells during aging results in an increased expression of proinflammatory cytokines through enhanced ROS production, which may contribute to the pathogenesis of inflammaging.

## 1 Introduction

Different immune cell subsets have a variety of metabolic demands and face various metabolic challenges. However, they all follow the basic principle of cell survival and function: maintaining homeostasis by metabolic adaptation (1). Once homeostasis is disturbed, such as during infection or in damaged tissue, a (usually) precise and timely process called inflammation is induced by signals released from the infected, dying (by any kind of destruction), or stressed cells and tissues (1). Inflammation also modulates cellular metabolic responses by providing energy and building blocks and thus the capability of immune cells to fulfill their function during inflammation. This process finally aims to re-establish homeostasis but can also be insufficient (in case of immunodeficiency of any reason), sub-clinically enhanced (e.g., during aging), or exacerbated, leading to (age-related) chronic inflammatory diseases and autoimmunity (e.g., rheumatoid arthritis: RA) (2).

In recent years, many countries have faced an expansion of the aging population due to increased life expectancy and low birth rates. The old-age dependency ratio (the ratio of persons aged 65 years and above to those aged 15-64 years) is expected to reach 53.5% in the European Union by 2060 (3). Aging influences the etiology of several economically significant diseases such as cancer, Alzheimer’s disease (AD), type 2 diabetes mellitus, stroke, and cardiovascular diseases age-dependent (4, 5). Age-related inflammation termed “inflammaging” represents the hub in their pathogenesis. It is characterized by elevated sub-clinical levels of proinflammatory cytokines such as IL-6, TNF-α, and IL-1. These cytokines play an essential role in developing most age-related diseases, and their expression strongly predicts morbidity and mortality in the elderly (6, 7).

CD4^+^ T cells are critical players in the adaptive immune system and are vastly involved in inflammaging (8). With increasing age, detrimental changes occur in the CD4^+^ T cell compartment. These include decreasing naive CD4+T cells and increasing different subsets of memory T cells (9, 10). Memory CD4^+^ T cells are relatively long-lived, quiescent adaptive immune cells with few biosynthetic needs. ATP is mainly generated by oxidative phosphorylation (OxPhos) required to maintain cells’ housekeeping functions (11, 12). Memory CD4^+^ T cells patrol the body and are activated upon antigen contact to clear pathogens. To complete this task, T cells adjust their metabolic profile in multiple ways: Firstly, they accelerate the metabolite flux through glycolysis, tricarboxylic acid (TCA) cycle, and OxPhos. Secondly, the proportion of energy supplied by glycolysis is augmented to meet the extensive demands of metabolic intermediates required for cell proliferation, differentiation, and the production of immunomodulatory molecules (1).

Fulop et al. (10) and Pawelec (13) reported that chronic CMV infections led to an accumulation of end-differentiated memory T cells also called senescent T cells (10, 13). This subset of memory T cells exhibits defective mitochondrial biogenesis and function, leading to an increased expression of proinflammatory cytokines (14). This so-called senescence-associated secretory phenotype (SASP) evolves in inflammaging (15). However, Burel et al. (9) did not detect an expansion of the senescent CD4^+^ T cell population with increasing age, while characteristic features of inflammaging were still observed in aging individuals.

As cellular metabolism modulates T cell function, it can be assumed that metabolic changes accompany the differentiation of memory CD4^+^ T cells into senescent CD4+ T cells and contribute to memory CD4^+^ T cells dysfunction during aging. Therefore, we hypothesized that metabolic reprogramming in memory CD4^+^ T cells might be an essential factor promoting immune cell dysfunction during aging, fueling the pathogenesis of age-related diseases.

## 2 Materials and methods

### 2.1 Cell purification and culture

Leukocyte depletion filters from anonymized healthy young donors (<35 years) and aged donors (>50 years) were provided by the Charité blood donation department with the approval of the Charité-Universitätsmedizin Ethics Committee and, according to the Helsinki Declaration (ethical approval EA1/207/17 and EA1/367/14). According to the modified manufacturer’s instructions, naive and memory CD4^+^ T cells were isolated from human PBMCs from leukocyte depletion filters by magnetically activated cell sorting (MACS). Memory CD4^+^ T cells were enriched by magnetic depletion of microbead-labeled naive T cells, CD8+ T cells, B cells, NK cells, γ/δ T cells, monocytes, DCs, granulocytes, platelets, and erythroid cells employing the human memory CD4^+^ T Cell Isolation Kit (Miltenyi Biotec). Naive CD4^+^ T cells were enriched by magnetic depletion of microbead-labeled memory T cells, CD8+ T cells, B cells, NK cells, γ/δ T cells, monocytes, DCs, granulocytes, platelets, and erythroid cells employing the human naive CD4^+^ T Cell Isolation Kit II (Miltenyi Biotec). To increase the purity of either memory or naive CD4^+^ T cells, a subsequent positive selection of anti-CD4 microbead-labeled cells (Miltenyi Biotec) was conducted. In short, to isolate CD4^+^ memory T cells, PBMCs were labeled with a cocktail of biotinylated CD45RA, CD8, CD14, CD16, CD19, CD56, CD36, CD123, anti-TCRγ/δ and CD235a (glycophorin A) antibodies and subsequently incubated with anti-biotin microbeads. The microbead-labeled cell suspension was pipetted onto an LS column (Miltenyi Biotec), and the flow-through comprising the unlabeled cells of interest was collected. To isolate CD4^+^ naive T cells, PBMCs were labeled with a cocktail of biotinylated CD45RO, CD8, CD14, CD16, CD19, CD56, CD36, CD123, anti-TCRγ/δ, and CD235a (glycophorin A) antibodies and subsequently incubated with anti-biotin microbeads. The microbead-labeled cell suspension was pipetted onto an LS column (Miltenyi Biotec), and the flow-through comprising the unlabeled cells of interest was collected. Next, incubation with human IgG (Baxter) was performed to block Fc receptors. After this step, anti-CD4 magnetic beads were added to the unlabeled either naïve or memory CD4^+^ T cells and washed by adding autoMACS^®^ Running Buffer (Miltenyi Biotec). Labeled cells were resuspended in buffer and placed onto an LS column (Miltenyi Biotec). The flow-through comprising the unlabeled CD4^-^ cells still present was discarded. The column was removed from the magnetic field, and labeled cells were flushed out of the column with the help of a plunger. Cells were cultured in RPMI 1640 medium (Thermo Fisher Scientific) with 5% human AB serum (Sigma Aldrich) and 1% penicillin/streptomycin (Thermo Fisher Scientific) at 37˚C in a 5% CO_2_ atmosphere.

### 2.2 Flow cytometry

#### 2.2.1 Surface marker staining

For surface molecule staining, cells were harvested, washed, and stained with a combination of monoclonal antibodies, including anti-human-CD3 (Pacific Blue, BD Biosciences, clone UCHT1), anti-human-CD4 (FITC, DRFZ, clone TT1), anti-human-CD45RO (PE, DRFZ, clone UCHL1), anti-human-CCR7 (PE-Cy7, BioLegend, clone G043H7) and anti-human-CD45RA (APC, DRFZ, clone 4G11).

#### 2.2.2 Intracellular cytokine staining

Before the intracellular cytokine staining, cells were stimulated with PMA and ionomycin for two hours, followed by a three-hour incubation period complemented with brefeldin A (BFA, Sigma Aldrich). Incubation was conducted by dissolving 1x 10^6^ cells/ml in RPMI 1640 medium (Thermo Fisher Scientific) with 5% human AB serum (Sigma Aldrich) and 1% penicillin/streptomycin (Thermo Fisher Scientific) at 37˚C in a 5% CO_2_ atmosphere Cells were collected, washed, fixed, permeabilized (Inside staining kit, Miltenyi Biotec) and stained with a combination of monoclonal antibodies including anti-human-IFNγ (PERCP-Cy5.5, Biolegend, clone 4SB3, Cat.# 502526), anti-human-IL-2 (APC-vio770, Miltenyi Biotec, clone N7.48 A, Cat.# 130-097-011), anti-human-IL-4 (PE, Miltenyi Biotec, clone 7A3-3, Cat.# 130-091-647), anti-human-IL-17A (FITC, Miltenyi Biotec, clone CZ8-23G1, Cat.# 130-094-520), and anti-human-TNF-α (PE-Vio770 (Miltenyi Biotec, clone cA2, Cat.# 130-096-755).

#### 2.2.3 Ki-67 staining

Ki-67 staining was performed after stimulation with anti-human CD3 and CD28 antibodies (T Cell TransAct™, Miltenyi Biotec) supplementation with IL-2 (PROLEUKIN^®^) and incubation for 72 h or 96 h in RPMI 1640 medium (Thermo Fisher Scientific) with 5% human AB serum (Sigma Aldrich), and 1% penicillin/streptomycin (Thermo Fisher Scientific) at 37˚C in a 5% CO_2_ atmosphere. Cells were harvested and fixed using 70% ethanol and stained with anti-human-Ki-67 (PerCP-Vio700, Miltenyi Biotec, clone REA183, Cat.# 130-100-292).

#### 2.2.4 CFSE staining

CFSE was applied at a final concentration of 2.5 μM (Sigma Aldrich). CSFE-labeled CD4^+^ memory T cells were analyzed by flow cytometry after 72 h and 96 h of stimulation with anti-human CD3 and anti-human CD28 antibodies as well as IL-2 and incubation in RPMI 1640 medium (Thermo Fisher Scientific) with 5% human AB serum (Sigma Aldrich), and 1% penicillin/streptomycin (Thermo Fisher Scientific) at 37˚C in a 5% CO_2_ atmosphere.

#### 2.2.5 iROS staining

To measure cellular oxidative stress, iROS were detected using 5 (and 6) - chloromethyl-2’,7’-dichlorodihydrofluorescein diacetate, acetyl ester (CM-H2DCFDA; Invitrogen GmbH, Karlsruhe, Germany). Cells were incubated 30 min in PBS (DRFZ, Berlin, Germany) with 5 µM CM-H2DCFDA, washed with glucose-free RPMI-1640, and incubated for 24 h in RPMI 1640 medium (Thermo Fisher Scientific) with 5% human AB serum (Sigma Aldrich), and 1% penicillin/streptomycin (Thermo Fisher Scientific) at 37˚C in a 5% CO_2_ atmosphere with and without stimulation using anti-human CD3 and CD28 antibodies (T Cell TransAct™, Miltenyi Biotec) and IL-2 (PROLEUKIN^®^). After incubation, cells were washed with PBS, and data were acquired using a BD FACSCanto™ II (BD Biosciences, San Jose, USA) and processed by FlowJo v7.6.5 (BD Biosciences, San Jose, USA).

#### 2.2.6 Mitochondrial content staining

To measure mitochondrial content, isolated memory Th cells were stained for 1 h using 1 μM MitoTracker™ Green FM (Thermo Fisher Scientific) after stimulation with anti-human CD3 and CD28 antibodies (T Cell TransAct™, Miltenyi Biotec) supplementation with IL-2 (PROLEUKIN^®^) and incubation for 24 h in RPMI 1640 medium (Thermo Fisher Scientific) with 5% human AB serum (Sigma Aldrich), and 1% penicillin/streptomycin (Thermo Fisher Scientific) at 37˚C in a 5% CO_2_ atmosphere. Staining was performed according to the manufacturer’s instructions.

#### 2.2.7 Flow cytometric acquisition

All stained cells were measured in a BD FACS Canto^®^II device with FACS^®^Diva software (BD Biosciences) and analyzed using FlowJo software (Tree Star). According to either FSC/SSC pattern, DAPI or PI staining, dead cells, and debris were excluded from the analysis.

### 2.3 ATP measurement

According to the manufacturer’s instructions, the ATP content of isolated memory Th cells was assessed using the CLS II KIT (Roche, Mannheim, Germany). The luminescence of all samples was quantified using the Synergy HT plate reader (BioTek, Bad Friedrichshall, Germany).

### 2.4 Immunofluorescence microscopy

Immunofluorescence staining was performed in a dark, humid chamber at RT. To measure mitochondrial content or cellular oxidative stress, cells were stained as described above and subsequently centrifuged onto a glass slide at 500 g for 1 minute with the help of a Cytospin 4 cytocentrifuge (Thermo Fisher Scientific). The cells were washed with PBS. High concentrated DAPI (10μg/mL in PBS/5% FCS, 10- minute incubation) was applied to detect the nuclei. After three 3-minute washing steps with PBS, images were acquired with an LSM 880 confocal laser scanning microscope (ZEISS, Germany, RRID: SCR_020925) and analyzed with ZEN (ZEISS).

### 2.5 Metabolic assays

The metabolic profile was evaluated in unstimulated naive/memory CD4^+^ T cells and memory CD4^+^ T cells after 24 h stimulation with anti-CD3 and anti-CD28 antibodies. Oxygen consumption rate (OCR) and Proton efflux rate (PER) were measured using an XFe-96 Extracellular Flux Analyzer (Agilent). Cells were seeded in a Seahorse 96-well plate (Agilent) at a concentration of 2.5 x 10^5^ cells/well (unstimulated cells) or 2 x 10^5^ (stimulated cells) cells/well (more than 5 duplicates) in RPMI-based medium (Agilent) supplemented with 10 mM D-glucose (Sigma Aldrich), 1 mM pyruvate (Sigma Aldrich), 2 mM glutamine (Sigma Aldrich) and 1 mM HEPES (Sigma Aldrich), adjusted to pH 7.4. OCR was measured under basal conditions and in sequential response to 2 μM oligomycin, 1.5 μM carbonylcyanide-4- (trifluoromethoxy) –phenylhydrazone (FCCP), and 0.5 μM Antimycin and Rotenone. PER was measured under basal conditions and in sequential response to 0.5 μM of Antimycin and Rotenone and 50 mM 2-DG (all from Sigma Aldrich). Data are depicted as cell numbers adjusted to the concentration of 2.5 x 10^5^ cells/well.

To facilitate reading and interpreting of metabolic data, Seahorse XF Cell Mito Stress Test assay kinetic profile, parameters, parameter equation, and their interpretation and Seahorse XF Glycolytic Rate assay kinetic profile, parameters, parameter equation, and their interpretation are provided as supplementary figures (**Supplementary Figures 2**, **3**).

### 2.6 Multiplex ELISA

Isolated memory Th cells were stimulated with anti-human CD3 and CD28 antibodies (T Cell TransAct™, Miltenyi Biotec) supplementation with IL-2 (PROLEUKIN^®^) with and without 1 µM MitoTEMPO (Sigma Aldrich) and incubated for 24 h in RPMI 1640 medium (Thermo Fisher Scientific) with 5% human AB serum (Sigma Aldrich), and 1% penicillin/streptomycin (Thermo Fisher Scientific) at 37˚C in a 5% CO_2_ atmosphere.

The supernatants were analyzed for IL-1RA, IL-2, IL-4, IL-5, IL-6, IL-8, IL-9, IL-10, IL-13, IL-17, GM-CSF, IFN-γ, IP-10, MCAF/MCP1 (CCL2), MIP-1a (CCL3), MIP-1b (CCL4), RANTES (CCL5), TNF-α, and VEGF in a Bio-Plex suspension system (Bio-Rad) according to the manufacturer’s instructions. Suspensions were diluted 1-to-1 in adequate buffers. The samples were measured on a Bio-Plex 200 (Bio-Rad).

### 2.7 Determination of glucose and lactate

Glucose and lactate levels were determined by the Institute of Laboratory Medicine and Pathobiochemistry (Charité, Berlin, Germany) according to standard operating procedures.

### 2.8 Estimation of ATP generation

Based on the assumption that the ATP output of glycolysis and OXPHOS is similar, that steady-state ATP is fed and consumed in the same range, and that the rate remains constant, we calculated the ATP produced by glycolysis:

*mitoOCR + glycoPER = mean of steady-state ATP measured (total ATP)*

*mitoOCR/glycoPER (basal) = mean of measured values from Seahorse™ experiments*

Note that glycolysis allows the synthesis of 2 moles of ATP per mole of glucose. In contrast, OXPHOS generates 36 moles of ATP per mole of glucose, and the oxidation of fatty acids generates about 106 moles of ATP per complete oxidation of one palmitate molecule. However, the rate of these different metabolic processes is not precisely known.

### 2.9 Statistics

Statistical analysis was performed using GraphPad^®^ Prism software (GraphPad Software, La Jolla, USA). Data were analyzed for normal distribution with the Shapiro–Wilk test or Kolmogorov-Smirnov-Test. Mann-Whitney U test and unpaired t-test (with Welch’s correction in data without equal variances) were applied for independent data. In contrast, dependent data were compared using paired t-test or Wilcoxon signed-rank tests. Multiple comparisons were analyzed by one- or two-way ANOVA, as indicated, with the Bonferroni multiple-comparison *post hoc* test. P values of <0.05 were considered statistically significant. The following symbols were used: * for P<0.05, ** for P<0.01, and *** for P<0.001.

## 3 Results

### 3.1 Memory CD4^+^ T cells exhibit a higher glycolytic and mitochondrial phenotype than naive CD4^+^ T cells

**Figure 1:**
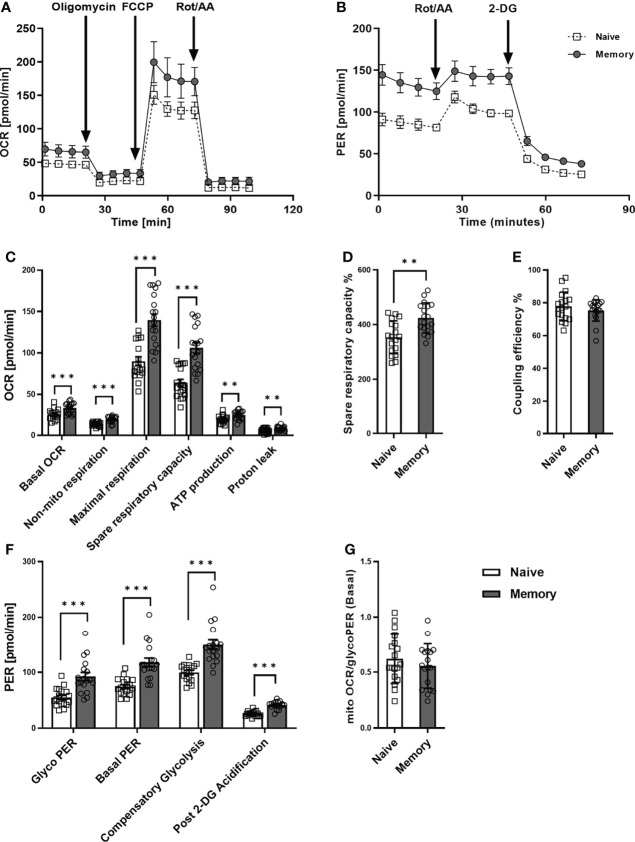
Mitochondrial profile and glycolytic function of naive and memory CD4^+^ T cells. Naive and memory CD4^+^ T cells were analyzed with regard to OCR or PER under basal conditions and after addition of the indicated reagents. Representative example of the Seahorse XF Cell Mito Stress Test kinetic profile and Glycolytic Rate kinetic profile of naive and memory CD4^+^ T cells **(A, B)**. Comparison of basal OCR, non-mito respiration, maximal respiration, spare respiratory capacity, ATP production, proton leak, spare respiratory capacity%, coupling efficiency between naive and memory CD4^+^ T cells **(C–E)**. Comparison of basal glycolysis, basal PER, compensatory glycolysis, post 2-DG acidification rate **(F)** and basal mitoOCR/glycoPER ratio **(G)** between naive and memory CD4^+^ T cells (mean ± SD; n = 18). A paired t test or Wilcoxon signed-rank test was performed after the normality test. **P<0.01; ***P<0.001. OCR, oxygen consumption rate; PER, proton efflux rate.

We first examined the metabolic phenotype of quiescent naive and memory CD4^+^ T cells (**Figures 1A,B**). To this end, the purity of naive and memory CD4^+^ T cells was verified by flow cytometry and generally exceeded 95% (**Supplementary Figure 1A**). Memory CD4^+^ T cells are quite heterogeneous, they include central memory and effector memory cells, which differ in phenotype, cytokine release, and replication capacity. Therefore, we initially analyzed our donors’ frequency of central memory and effector memory cells. We found no differences in the frequencies of these populations between the two groups surveyed (**Supplementary Figure 1B**). The basal oxygen consumption rate (OCR) of memory CD4^+^ T cells was significantly higher than that of CD4^+^ naive T cells (**Figure 1C**). Compared to CD4^+^ naive T cells, memory CD4^+^ T cells demonstrated a significantly greater increase in maximal respiration and spare respiratory capacity (SRC), indicating mitochondrial reserve capacity. The percentage of SRC (**Figure 1D**) was also significantly higher in memory CD4^+^ T cells than in CD4^+^ naive T cells. In addition, memory CD4^+^ T cells also demonstrated higher values for (i) non-mito OCR, (ii) ATP production, and (iii) proton leak as compared to naive CD4^+^ T cells. However, the CD4^+^ T cell subsets showed similar coupling efficiency (**Figure 1E**).

Concerning glycolytic function, we observed significantly higher basal glycolysis rates (glycoPER) in memory CD4^+^ T cells than in naive CD4^+^ T cells (**Figure 1F**). Besides, the compensatory glycolysis rate of memory CD4^+^ T cells significantly exceeded that of naive CD4^+^ T cells. Moreover, basal PER and the post-2-DG acidification rate were substantially higher in memory CD4^+^ T cells than in CD4^+^ naive T cells. Conversely, we discovered no statistical difference between the two groups’ ratios of basal mitoOCR/glycoPER (**Figure 1G**).

In summary, higher basal respiration rates, SRC, glycolysis, and compensatory glycolysis were demonstrated in memory CD4^+^ T cells compared to naive CD4^+^ T cells, whereas mitoOCR/glycoPER ratios were alike. These findings indicate that memory CD4^+^ T cells generally exhibit higher glycolytic and mitochondrial fluxes than naive CD4^+^ T cells.

### 3.2 Quiescent memory CD4^+^ T cells of aged donors demonstrated lower basal glycolysis and compensatory glycolysis and higher than those from young donors in a quiescent state

**Figure 2:**
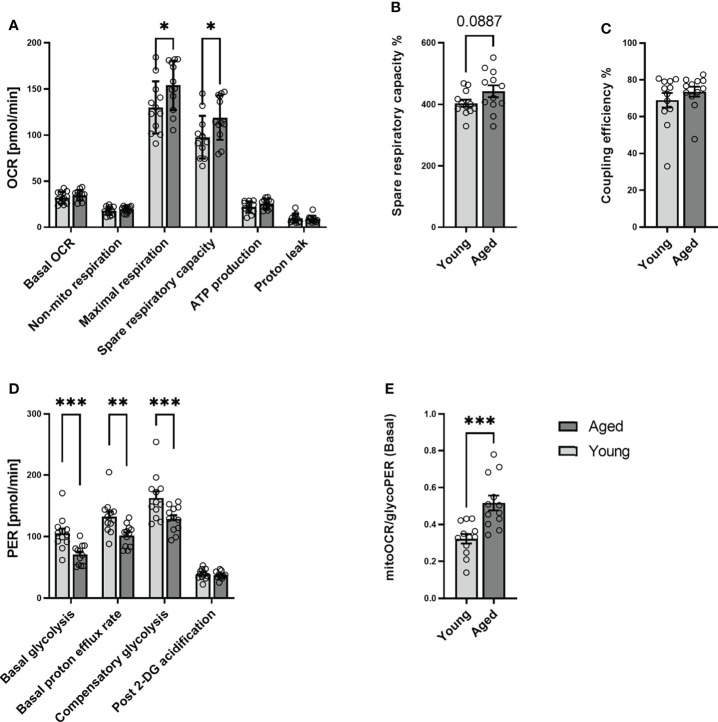
Mitochondrial profile and Glycolytic profile of memory CD4^+^ T cells in young and aged donors. Comparison of basal OCR, non-mito respiration, maximal respiration, spare respiratory capacity, ATP production, proton leak, spare respiratory capacity%, and coupling efficiency in memory CD4^+^ T cells from young donors and aged donors **(A–C)**. Comparison of basal glycolysis, basal PER, compensatory glycolysis, post 2-DG acidification rate, and basal mitoOCR/glycoPER ratio in memory CD4^+^ T cells from young and aged donors **(D, E)** (mean ± SD; n = 12). In A and D, multiple comparisons were analyzed by two-way ANOVA, with the Bonferroni multiple-comparison *post hoc* test. In B, C, E, a Mann- Whitney test was performed. *P<0.05, **P<0.01; ***P<0.001. OCR, oxygen consumption rate; PER, proton efflux rate.

To further investigate the metabolic phenotype of quiescent T cells young and aged, we analyzed memory CD4^+^ T cells from 12 young and 12 aged individuals who were matched by sex (**Table 1**). Mitochondrial and glycolytic profiles of naive CD4^+^ T cells did not differ significantly between young and aged donors (**Supplementary Figure 4**). In contrast, focusing on memory CD4^+^ T cells, we observed higher maximal respiration and a higher spare respiratory capacity in the aged group (**Figures 2A–C**). Additionally, memory CD4^+^ T cells from old donors demonstrated lower basal glycolysis rates than young donors (**Figure 2D**). Basal PER is composed of mitochondrial acidification and basal glycolysis. Mitochondrial respiration is accompanied by the generation of CO_2,_ which is transported into the cytoplasm, enhancing acidification. Aged individuals presented lower levels of basal PER in comparison to young donors. As both groups showed a similar basal OCR, the lower basal PER is explained by the lower basal glycolysis in the elderly. Additionally, lower rates of compensatory glycolysis were identified in memory CD4^+^ T cells from aged donors. In line with all these findings, memory CD4^+^ T cells from aged donors demonstrated a significantly higher ratio of basal mitoOCR/glycoPER (**Figure 2E**).

**Table 1:** Characteristics of young and aged donors as analyzed using metabolic measurements.

Data-sets per figure	CD4^+^ T cell subtypes	Sample numbers	Donors*				Assays
			male young	male aged	female young	female aged	
**Figure 1**	naive	18	6	6	3	3	Seahorse™ Mito Stress Test, Seahorse™ Glycolytic Rate Assay
**Figure 1**	memory	18	6	6	3	3	Seahorse™ Mito Stress Test, Seahorse™ Glycolytic Rate Assay
**Figure 2**	memory	24	9	9	3	3	Seahorse™ Mito Stress Test, Seahorse™ Glycolytic Rate Assay
**Figure 3**	memory	24	12	12			Seahorse™ Mito Stress Test, Seahorse™ Glycolytic Rate Assay
**Figure 3**	memory	12	6	6			ATP level
**Figure 3**	memory	18	9	9			glucose conc., and lactate conc.
**Figure 4**	memory	12	6	6			Mitochondrial content and intracellular ROS production
**Figure 5B**	memory	16	8	8			Proliferation
**Figure 5C**	memory	12	6	6			Ki-67 staining
**Figure 6**	memory	14	7	7			IC staining of cytokines
**Figure 7**	memory	12	6	6			Secreted cytokines and mitoTEMPO
**Supplementary Figure 1**	memory	18	6	6	3	3	CCR7 staining
**Supplementary Figure 4**	naive	18	6	6	3	3	Seahorse™ Mito Stress Test, Seahorse™ Glycolytic Rate Assay
**Supplementary Figure 5**	naive	12	6	6			Seahorse™ Mito Stress Test, Seahorse™ Glycolytic Rate Assay
**Supplementary Figure 5**	memory	12	6	6			Seahorse™ Mito Stress Test, Seahorse™ Glycolytic Rate Assay
**Supplementary Figure 6**	naive	12	6	6			Seahorse™ Mito Stress Test, Seahorse™ Glycolytic Rate Assay
**Supplementary Figure 7**	memory	16	8	8			Seahorse™ Mito Stress Test, Seahorse™ Glycolytic Rate Assay

* young: range of 20 – 35 years; aged: range of 51 – 69 years.

We interpret these results that aging affects neither the metabolic phenotype of quiescent naive CD4^+^ T cells nor the basal mitochondrial function of quiescent memory CD4^+^ T cells. At the same time, it increases mitochondrial spare respiratory capacity and causes a decline in the ability and reserve capacity to utilize glycolysis.

To exclude deviations from sex hormones, differences in sex hormone receptor expression, and the different responsiveness of male and female T cells about proliferation and Th1 response (16), we restricted the analysis of the above metabolic data to male individuals (**Supplementary Figures 5**–**7**). Therefore, the enrolment for the following metabolic and functional experiments was restricted to male donors.

### 3.3 Stimulated memory CD4^+^ T cells from aged donors demonstrated higher spare respiratory capacity than those from young donors

**Figure 3:**
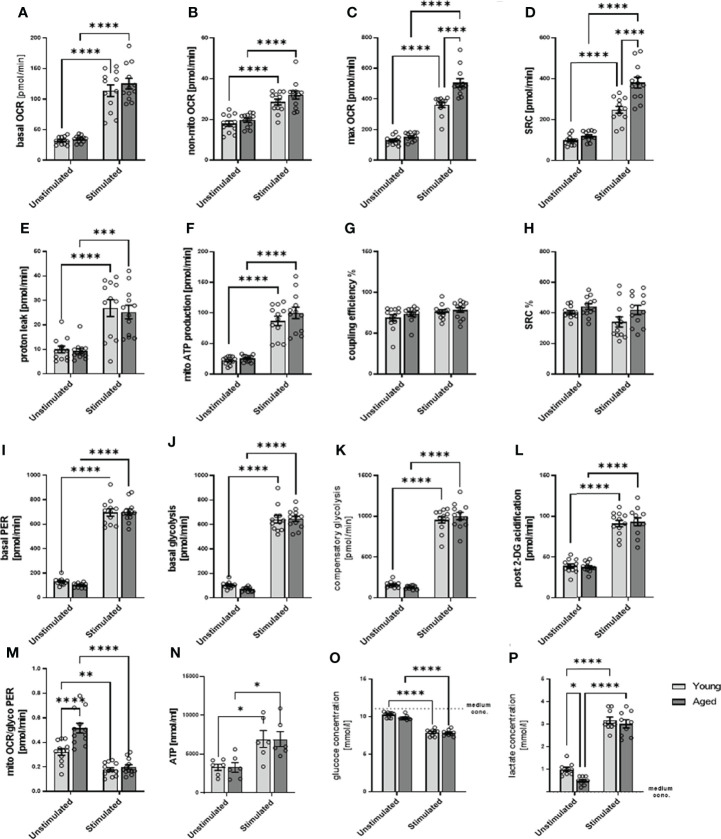
Mitochondrial profile, glycolytic profile, steady-state ATP level, glucose consumption, and lactate secretion of stimulated/unstimulated memory CD4^+^ T cells in young and aged male donors. Comparison of basal OCR **(A)**, non-mito respiration **(B)**, maximal respiration **(C)**, spare respiratory capacity **(D)**, proton leak **(E)**, ATP production **(F)**, coupling efficiency **(G)**, and spare respiratory capacity% **(H)** in stimulated/unstimulated memory CD4+ T cells from young and aged donors. Comparison of basal PER **(I)**, basal glycolysis **(J)**, compensatory glycolysis **(K)**, post-2-DG acidification rate **(L)**, and basal mitoOCR/glycoPER ratio **(M)** in stimulated/unstimulated memory CD4^+^ T cells from young and aged donors (mean ± SEM; n = 12). **(N)** Steady-state ATP content of stimulated/unstimulated memory CD4^+^ T cells from young and aged donors after 24 h was measured by bioluminescence (mean ± SEM; n = 6). **(O)** Comparison of the reduction in medium glucose concentration as a measure of glucose consumption and **(P)** the increase in medium lactate concentration as a measure of glycolytic lactate production in stimulated/unstimulated memory CD4^+^ T cells from young and aged donors (mean ± SEM; n=9). **(A–P)** Multiple comparisons were analyzed by two-way ANOVA with the Bonferroni multiple-comparison *post hoc* test. *P<0.05, **P<0.01; ***P<0.001, ****P<0.0001. OCR, oxygen consumption rate; PER, proton efflux rate.

After stimulation, memory CD4^+^ T cells augmented their metabolic activity (**Figures 3A**–**M**), on both mitochondrial and glycolytic activity, as seen in, e.g., basal OCR and mitochondrial ATP production but also and even more pronounced in basal glycolysis, basal PER, compensatory glycolysis, post-2-DG acidification in both, stimulated memory CD4^+^ T cells from young and aged donors. Of note, stimulated memory CD4^+^ T cells from aged donors presented statistically significant higher maximal OCR, spare respiratory capacity, and a basal mitoOCR/glycoPER (**Figures 3C**, **D**, **M**) and numerically higher basal OCR, non-mitochondrial OCR, and ATP production (**Figures 3A**, **B**, **F**) indicating higher mitochondrial activity. However, both groups’ steady-state ATP levels remained similar (**Figure 3N**).

Since we analyzed total ATP levels at steady state, we estimate that ATP produced by glycolysis according to PER is 76% in unstimulated memory CD4^+^ T cells from young donors. According to OCR, ATP produced by OXPHOS is 24% of total ATP. In unstimulated memory CD4^+^ T cells from older donors, the proportion of ATP produced by glycolysis is 66%, and the proportion of ATP produced by OXPHOS is 34% of total ATP, according to OCR. In stimulated memory CD4^+^ T cells from young donors, ATP produced by glycolysis increased to 85%, and ATP produced by OXPHOS was 15% of total ATP. In contrast, in older donors, ATP produced by glycolysis is 83% and ATP produced by OXPHOS is 17%. However, this calculation is based on the assumption that the ATP output of glycolysis and OXPHOS is similar, that steady-state ATP is fed and consumed in the same range, and that the rate remains constant. Note that the following has to be considered: glycolysis allows the synthesis of 2 moles of ATP per mole of glucose, whereas OXPHOS generates 36 moles of ATP per mole of glucose, and the oxidation of fatty acids generates about 106 ATP per complete oxidation of one molecule of palmitate although the rate of these different metabolic processes is not precisely known (12).

Moreover, stimulated memory CD4^+^ T cells revealed an increased glucose consumption than unstimulated cells. Still, we observed no statistical difference between young and aged groups, neither in quiescent nor stimulated states (**Figure 3O**). The stimulation-induced increase in glucose consumption is accompanied by an increased lactate production (**Figure 3P**). However, under quiescent conditions, memory CD4^+^ T cells from aged donors show a lower lactate production than memory CD4^+^ T cells from young donors.

Since stimulated memory CD4^+^ T cells from aged donors increased their spare respiratory capacity to a greater extent than young donors, let us suggest that they own a higher capability to respond to an energetic demand which can be achieved by several mechanisms. These include a higher number of mitochondria and an increasing number of mitochondrial cristea or the electron transport chain complexes. Because of the lower basal glycolysis and the higher stimulation-induced spare respiratory capacity, we assumed a higher number of mitochondria in memory CD4^+^ T cells from aged donors, which may fail to contribute to higher steady-state ATP levels but are capable of compensating lower glycolytic rate.

### 3.4 Memory CD4^+^ T cells from aged individuals show a higher mitochondrial content and intracellular reactive oxygen species production than young donors

**Figure 4:**
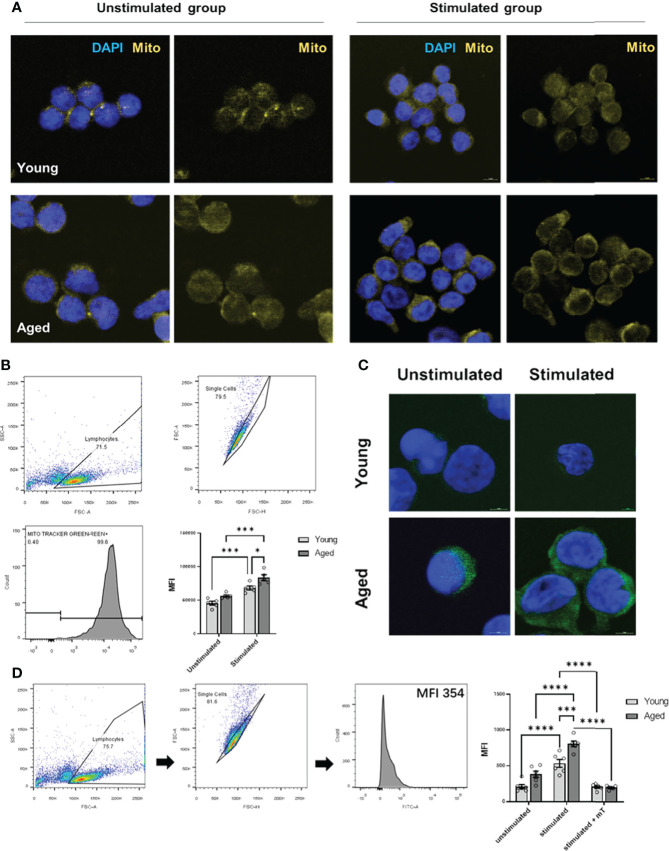
Mitochondrial content and intracellular ROS production of stimulated/unstimulated memory CD4^+^ T cells in young and aged male donors. **(A)** Representative images of mitochondrial content (yellow) in stimulated/unstimulated memory CD4^+^ T cells in young and aged male donors after 24 h visualized by immunofluorescence microscopy. Cells were stained using Mitotracker™ (yellow) and DAPI (blue). The scale bar represents 10 μm. **(B)** Representative example of single-cell mitochondrial content Mitotracker™ staining using flow cytometry (including gating) in stimulated and unstimulated memory CD4^+^ T cells and comparison of single-cell mitochondrial content *via* MFI in stimulated/unstimulated memory CD4^+^ T cells from young and aged donors (mean ± SEM; n = 6). **(C)** Representative images of intracellular ROS production (green) in stimulated/unstimulated memory CD4^+^ T cells in young and aged male donors after 24 h visualized by immunofluorescence microscopy. Cells were stained using DCF (green) and DAPI (blue). The scale bar represents 10 μm. **(D)** Representative example of single-cell intracellular ROS staining using flow cytometry (including gating) in stimulated and unstimulated memory CD4^+^ T cells and comparison of single-cell intracellular ROS *via* MFI in memory CD4^+^ T cells from young and aged donors were stimulated, stimulated and treated with mitoTEMPO (mT), and left unstimulated/untreated (mean ± SEM; n = 6). Multiple comparisons were analyzed by two-way ANOVA with the Bonferroni multiple-comparison *post hoc* test. *P<0.05, ***P<0.001, ****P<0.0001 **(C, D)**.

As memory CD4^+^ T cells from aged individuals without and with TCR activation showed a greater respiratory reserve (**Figures 2A**, **3D**) than young individuals, we analyzed the mitochondrial content. Indeed, we observed a higher number of mitochondria/mitochondrial content in activated memory CD4^+^ T cells from elderly individuals (**Figure 4A**, **B**). Moreover, an age-related increase in mitochondrial content and reserve capacity was associated with increased intracellular reactive oxygen species (iROS) production (**Figures 4C**, **D**).

### 3.5 Memory CD4^+^ T cells from aged donors secreted higher amounts of cytokines than those from young donors

**Figure 5:**
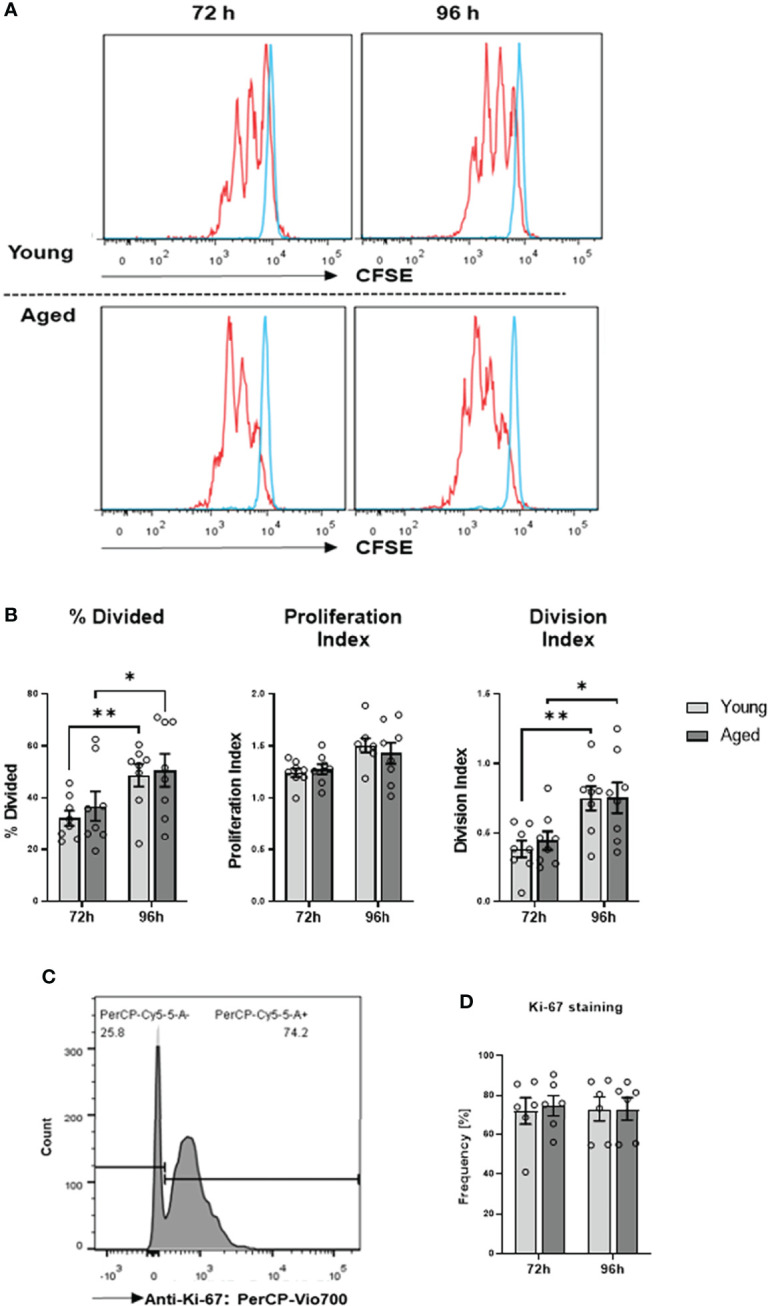
Proliferation of memory CD4^+^ T cells. **(A)** Representative example of flow cytometric analysis for CFSE stained memory CD4^+^ T cells from young and aged donors after 72 h and 96 h of TCR stimulation. **(B)** Comparison of division index, proliferation index, and percentage divided cells between memory CD4^+^ T cells from young donors and aged donors after 72 h and 96 h of TCR stimulation (mean ± SEM; n = 8 per group and time point). **(C)** Representative example of Ki-67 positive memory CD4^+^ T cells acquired by flow cytometry. **(D)** Comparison of Ki-67 positive memory CD4^+^ T cells from young donors and aged donors after 72 h and 96 h of TCR stimulation (mean ± SEM; n = 6 per group per group and time point). Multiple comparisons were analyzed by two-way ANOVA with the Bonferroni multiple comparison post hoc test. *P<0.05, **P<0.01 **(B, D)**.

We have demonstrated previously that aberrant high iROS negatively impacts T cell proliferation (17). Therefore, we investigated whether the transition of the memory CD4^+^ T cell phenotype during aging affects cell function. To this end, we conducted cell proliferation and cytokine production/secretion assays. Cell proliferation was examined by stimulating memory CD4^+^ T cells with anti-CD3/28 antibodies for 72h and 96h, followed by CSFE and Ki-67 measurements. We observed no significant differences in cell division and proliferation indices, the percentage of divided cells, and Ki-67 expression (**Figures 5A**–**D**).

**Figure 6:**
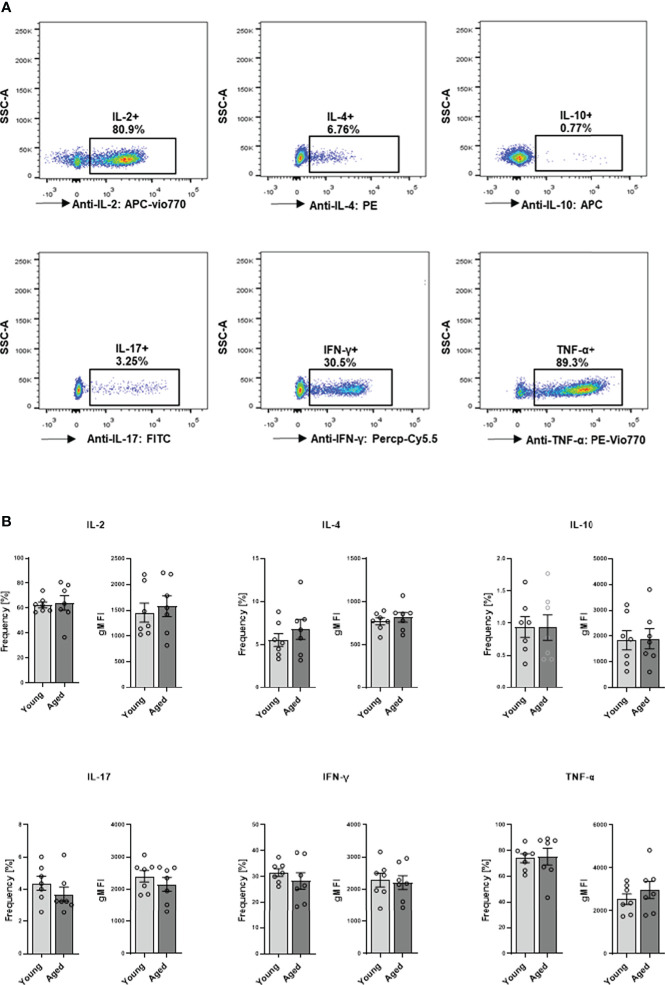
Capacity of cytokine production in memory CD4^+^ T cell. Cells were activated with PMA/ionomycin for 5 h, adding BFA for the last 3 hours for intracellular cytokine expression. **(A)** Representative example of cytokine expression in memory CD4^+^ T cells from young and aged donors after stimulation with PMA/ionomycin. **(B)** Frequencies in % and geometric mean fluorescence intensity (gMFI) of IL-2, IL-4, IL-10, IL-17, IFN-γ, and TNF-α producing memory CD4^+^ T cells (mean ± SEM; n = 7).

We proceeded to explore cytokine production and secretion of memory CD4^+^ T cells. Intracellular cytokine expression of memory CD4^+^ T cells was analyzed after stimulation with PMA and ionomycin. Flow cytometric analysis revealed no significant differences between young and aged donors in the frequency of IL-2, IL-4, IL-10, IL-17, IFN-γ, and TNF-α expressing cells or analyzed cytokine amount per cell as indicated by gMFI (**Figures 6A**, **B**).

**Figure 7:**
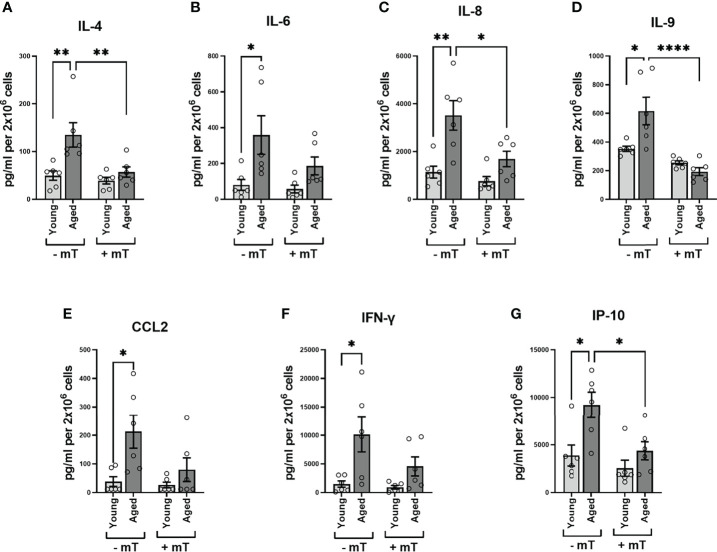
Cytokine secretion of memory CD4^+^ T cells. **(A–G)** Analysis of cytokine secretion in the supernatant of memory CD4^+^ T cells 24 h after TCR stimulation without and with mitoTEMPO(mT)-mediated reduction of cytokine secretion (mean ± SEM; n = 8). Mann-Whitney test was performed. *P<0.05, **P<0.01.

As PMA and ionomycin are potent stimulators of cytokine expression, the values referred to above might represent maximum cytokine production levels (the capacity to produce cytokines), neglecting possible differences regarding the kinetics of cytokine expression or signaling cascades involved in these two groups. Since we observed metabolic differences after TCR stimulation, we examined the cytokine amount secreted in the supernatants after 24h TCR stimulation. The secretion of IL-4, IL-6, IL-8, IL-9, IFN-γ, interferon γ-induced protein 10kDa (IP-10), monocyte chemotactic protein 1 (MCAF; MCP1; CCL2) in the supernatants of memory CD4^+^ T cells from aged donors significantly exceeded that measured in supernatants of memory CD4^+^ T cells from young donors (**Figures 7A**–**G**). Moreover, when interfering with mitochondrial ROS production using mitoTEMPO (mT), we observed a substantial reduction of secreted cytokine levels in the supernatants of memory CD4^+^ T cells from aged donors (**Figures 7A**–**G**), indicating a pivotal role for mitochondrial ROS production in impacting the TCR-mediated cytokine response in memory CD4^+^ T cells from elderly individuals.

## 4 Discussion

Although both memory and naive T cells physiologically tend to reside in a quiescent state, the recall response of memory cells to antigens is much quicker and more robust. This distinct behavior is supported by a unique metabolic phenotype, including greater mitochondrial mass, higher glycolytic reserve, and higher levels of cytoplasmic glyceraldehyde 3 phosphate dehydrogenase (GAPDH) (18–21).

In this study, we observed substantially higher basal respiration rates and SRC in CD4+ memory T cells than in naive CD4^+^ T cells. In line with our results, the enhancement of SRC by greater mitochondrial mass has been shown in previous studies (18, 21). Our finding that memory CD4^+^ T cells possess greater basal and compensatory glycolysis capacities also conforms with previous studies on human CD4^+^ effector memory T cells (21).

In principle, these distinctions of immune cell metabolism in different T cell subsets are known, but the impact of aging on the metabolic phenotype has not been extensively examined. While Burel et al. (9) observed a reduction in the population of human CD4^+^ naive T cells with increasing age, we observed stable metabolic parameters comparing young and aged donors in our study. Recently, a study performed on stimulated naive murine CD4^+^ T cells revealed lower OCR, maximal respiration, and glycolysis rates in old subjects than in the young reference group due to a specific deficit in the induction of enzymes one-carbon metabolism (22).

In contrast to human CD4^+^ naive T cells, aging impaired the glycolysis profile of memory CD4^+^ T cells. Here, we first demonstrate that memory cells from aged donors present decreased glycolysis, compensatory glycolysis, and a higher basal mitoOCR/glycPER while maintaining, if not increasing mitochondrial respiratory function in a quiescent state, leading to a higher proportion of ATP generated by OXPHOS. As the exclusion of female donors from our original dataset did not alter the results, it can be assumed that, contrary to age, sex did not significantly affect the metabolism of memory CD4^+^ T cells.

Besides pyruvate glycolysis generates several metabolites that are used to fuel the pentose phosphate pathway (PPP) or serve as building blocks of nucleotides, amino acids, and lipids to support cell growth, differentiation, proliferation, and production of effector molecules such as cytokines, chemokines and adhesion molecules (23). While the glycolysis rate was lower in the aged group, glucose uptake did not differ between both groups. Glucose usage in memory cells from aged donors predominantly sustains nucleotide, amino acid, and lipid synthesis and the PPP and also maintenance of iROS homeostasis instead of serving as a substrate for the production of the glycolysis end-product pyruvate, which in turn is either metabolized to lactate secreted to the external matrix (measure by Seahorse, indicated by glycoPER) or directly transferred to the mitochondria (12, 24).

Effector T cells face increased energetic demands to fulfill their effector functions during activation or inflammation primarily covered by glycolysis. As this can also be assumed valid for the process of inflammaging in at least effector memory T cells, we hypothesized that the glycolysis rate in the memory cell population from aged donors would significantly exceed that of young donors. However, we did not observe any statistical differences in basal or compensatory glycolysis after activation when comparing the young and aged groups. This finding was confirmed very recently (25).

Interestingly, the aged group showed a higher SRC (before and after stimulation) than the young group. Memory T cells exhibit an enhanced SRC facilitated by a greater mitochondrial mass (18, 21). The SRC represents the mitochondrial reserve capacity for oxygen usage and energy generation. A study on CD8^+^ T cells revealed a third vaccination boost to cause higher basal SRC supported by a further increase in mitochondrial mass than the first immunization (26). Greater mitochondrial mass and SRC equip T memory cells with the metabolic capacity to proliferate quickly and enhance their cytokine production. Thus, they have a bioenergetic advantage underlying their rapid recall ability. Similarly, memory CD4^+^ T cells in aged donors represent more experienced cells and do therefore exhibit greater SRC due to increased mitochondrial biogenesis upon activation than those from young donors. Our data underscore this mechanism. In contrast to our study presented here, Yanes et al. observed a higher basal OCR and ATP coupled OCR but no differences in SRC between the young (<35 years) and the aged (>65 years) (25). Although a higher mitochondrial mass may provide an evolutionary advantage, one has to look at the other side of the coin, which is the synthesis of higher amounts of intracellular mitochondrial ROS. iROS has been demonstrated to interfere with immune functional processes, as we and others could show previously (17, 27).

Thus, we aimed to determine whether the higher SRC, higher mitochondrial content (higher number of active ATPases or electron transport chain complexes in a higher number of mitochondrial cristae or a higher number of mitochondria), or even iROS contribute to the process of inflammaging in memory CD4^+^ T cells. In this study, neither cell proliferation nor intracellular cytokine expression capacity of memory Th cells was altered by aging. However, elevated levels of common proinflammatory cytokines (IFN-γ, IL-4, IL-6, IL-8, IL-9), including the Th1-type cytokine IFN-γ, Th2-type cytokine IL-4 and Th9-type cytokine IL-9 well as chemokines (IP-10, MCP1) were secreted at significantly higher levels by memory CD4^+^ T cells from aged donors 24 h post-TCR stimulation. Using the well-known mitochondrial ROS scavenger mitoTEMPO, we demonstrated that mitochondrial iROS drive enhanced cytokine secretion, finally contributing to the age-related increase of T cell-mediated inflammation (28).

ROS are well-known to cause collateral damage to macromolecules, and cellular organelles, eventually leading to uncontrolled inflammation and tissue damage once the quality control process of mitochondria is disturbed and coupling efficiency of the electron transport is leaky (29, 30). However, iROS from OXPHOS and cytoplasmic ROS from NADPH oxidases (NOXs) act as signaling molecules required to drive T cell activation, proliferation, and differentiation (17, 27). TCR ligation increases the production of ROS and initiates signaling cascades and activation of transcriptional factors, including the nuclear factor of activated T cells (NFAT), activator protein 1 (AP-1), and nuclear factor of kappa light chain enhancer in B cells (NFκB) leading to enhanced production of pro-inflammatory cytokines and chemokines and IL-2 mediated proliferation (30). This seems specific for the aforementioned pathways and may explain why some cytokines are affected while others are not. Previous studies have demonstrated that aging results in T cell dysfunction, leading to enhanced production of pro-inflammatory cytokines and chemokines, including TNF-α and IFN-γ, in the periphery and the bone marrow and involving ROS-mediated cell damage (14, 31). Although Albertini et al. observed a decrease in IFN-γ producing activated/memory T cells and an increase in IL-4 positive cells in the activated/memory CD4+ subset from nonagenarians (32), earlier reports demonstrated an increase in type 1 (IL-2, IFN-γ, TNF-α) and type 2 (IL-4, IL-6, IL-10) cytokines within CD8+ subsets in aged subjects (33). Since our study includes subjects with very little information about the donors and, moreover, the donors in the “old” group were not particularly old, one must consider explanations that offer an alternative answer for these pronounced functional differences in both groups studied. In particular, although somewhat speculative, chronic CMV infection may contribute to an accumulation of senescent dysfunctional T cells with defective mitochondrial biogenesis and function, leading to increased expression of proinflammatory cytokines (10, 13, 14).

IL-6 acts as a critical marker of inflammation as well as a feature of aging and plays a role in the pathophysiological mechanisms driving age-related diseases (e.g., osteoporosis, heart failure, AD, the decline in cognitive performance, and community-acquired pneumonia requiring hospitalization) (34–38). Moreover, an elevated IL-6 level is also associated with functional decline in aged individuals, causing poor physical performance and muscle weakness (39). Recently, the role of IL-6 in inflammaging has been emphasized by including the cytokine in an inflammation index score that was able to predict 10-year mortality in older adults (40).

Production of IL-8, sometimes called chemokine (C-X-C motif) ligand 8 or CXCL-8, is a hallmark of inflammation and responsible for neutrophil recruitment and activation and in several age-related diseases such as osteoporosis, Alzheimer’s disease (AD), or rheumatoid arthritis (41–43). In line with our findings, Mariani et al. reported a higher secretion of IL-8 aging by T lymphocytes from old subjects (>90 years) as compared to young subjects (<36 years) following stimulation (44).

Furthermore, we identified the chemokine interferon γ-induced protein 10kDa (IP-10) to be secreted more in aged subjects than in young ones. IP-10, also termed C-X-C motif chemokine 10 (CXCL10), belongs to the CXC chemokine family and participates in leukocyte trafficking and activation of immune cells as a pro-inflammatory molecule. High levels of IP-10 are associated with AD, age-related macular degeneration (AMD), infectious diseases, autoimmune diseases, and tumor development (45–48).

MCAF, known as monocyte chemoattractant protein-1 (MCP-1), is also a member of the CC chemokine subfamily. MCAF is critical in recruiting immune cells, such as monocytes, memory T cells, and DCs, to inflammatory sites (49). Like IP-10, MCAF is involved in age-related diseases, such as diabetes, diabetic nephropathy, atherosclerosis, and tumors (49).

The proinflammatory cytokine IL-9 is mainly secreted by Th9 cells. Besides a central role in the pathogenesis of allergic inflammation, IL-9 contributes to the immune response against extracellular parasites and other pathogens. Still, its part in inflammaging needs further addressed (50).

Aging is associated with elevated sub-clinical levels of proinflammatory cytokines such as IL-6, TNF-α, and IL-1 (6, 7). In particular, memory T cells exhibit defective mitochondrial biogenesis and function leading to an increased expression of proinflammatory cytokines the so-called senescence-associated secretory phenotype (SASP) (14, 15). Here we demonstrate that metabolic reprogramming in human memory Th cells during aging results in an increased expression of proinflammatory cytokines through enhanced mitochondrial ROS production. Apart from ROS generation, mitochondrial metabolism but not glycolysis has been well documented to be highly modulated by Calcium in CD4+ T-cells, primarily upon TCR stimulation and *vice versa* (51–53). Recruitment of mitochondria to the forming immunological synapse maximizes the efficiency of calcium influx through ORAI channels while it decreases calcium clearance by plasma membrane calcium ATPases being essential for calcium-dependent NFAT activity and subsequent T-cell activation (51–53).

Dysregulation in Ca^2+^ homeostasis is a hallmark of many age-related diseases and age-related decreases in Ca^2+^ signaling have been linked to dysfunction in aged lymphocytes (54). Angenendt et al. recently reported reduced calcium influx through ORAI channels and increased Ca^2+^ clearing rates by plasma membrane calcium ATPases in CD8^+^ T cells of elderly mice reducing CTL cytotoxicity (55). However, the role of an age-dependent reprogramming of mitochondrial metabolism and its impact on Ca^2+^ homeostasis and signaling during lymphocyte aging needs to be elucidated.

## 5 Conclusion

This study used leukocyte depletion filters from healthy donors supplied by the Charité blood donation department. Due to data protection regulations, information on the donors’ body mass index (BMI), ethnicity, and disease history were not available. We allocated the donors to two different groups according to their chronological age, disregarding their biological age. Since there is considerable heterogeneity concerning the prevalence of age-related diseases among the two groups of chronologically aged individuals, we predefined a wide age range (more than 30 years) to examine general trends in metabolic and functional differences between the aged and young groups.

Importantly, we purified memory CD4^+^ T cells with the help of microbeads, including anti-CD45RA depletion antibodies. Therefore, CD45RA re-expression memory CD4^+^ T cells, also called TEMRA cells, were excluded from our memory CD4^+^ T cell population. This was also confirmed by flow cytometry. Consequently, we used non-senescent memory CD4^+^ T cells in our study. This subset presented distinct metabolic profiles in cells isolated from elderly individuals compared to the younger reference group, both in quiescent and activated states. This phenotype was accompanied by enhanced cytokine expression. It follows that metabolic changes in memory CD4^+^ T cells precede the manifestation of senescent markers during human aging due to enhanced mitochondrial ROS production. Thus, the substantial amount of proinflammatory cytokines secreted by non-senescent memory CD4^+^ T cells during aging may also advance the process of inflammaging.

## Data availability statement

The raw data supporting the conclusions of this article will be made available by the authors, without undue reservation.

## Ethics statement

The studies involving human participants were reviewed and approved by Charité-Universitätsmedizin Ethics Committee. The patients/participants provided their written informed consent to participate in this study.

## Author contributions

TG designed the study. TG and YC conceived and supervised the research. YC, YY, P-LK, PL, MP, AD, LE, PH, TB, and FB collected, analyzed, and interpreted the experimental data. All authors contributed to writing or reviewing the manuscript and final approval. All authors contributed to the article and approved the submitted version.

## Funding

This work was supported by the China Scholarship Council (CSC, No. 201606230248) to YC and the German Research Foundation (DFG, No. 353142848) to TG. AD was supported by the Studienstiftung des deutschen Volkes and by the Joachim Herz Foundation (Add-on Fellowship 2020). We acknowledge support from the German Research Foundation (DFG) and the Open Access Publication Fund of Charité – Universitätsmedizin Berlin.

## Conflict of interest

The authors declare that the research was conducted without any commercial or financial relationships that could be construed as a potential conflict of interest.

## Publisher’s note

All claims expressed in this article are solely those of the authors and do not necessarily represent those of their affiliated organizations, or those of the publisher, the editors and the reviewers. Any product that may be evaluated in this article, or claim that may be made by its manufacturer, is not guaranteed or endorsed by the publisher.
