# Supplementary material for: Age-related increase of mitochondrial content in human memory CD4+ T cells contributes to ROS-mediated increased expression of proinflammatory cytokines
Source: Front Immunol. 2022 Jul 22;13:911050. doi: 10.3389/fimmu.2022.911050 (PMC9353942; doi:10.3389/fimmu.2022.911050)
Supplement: Supplementary file 1 [file DataSheet_1.pdf]

Supplementary Material

1 Supplementary Figures and Tables

1.1 Supplementary Figures

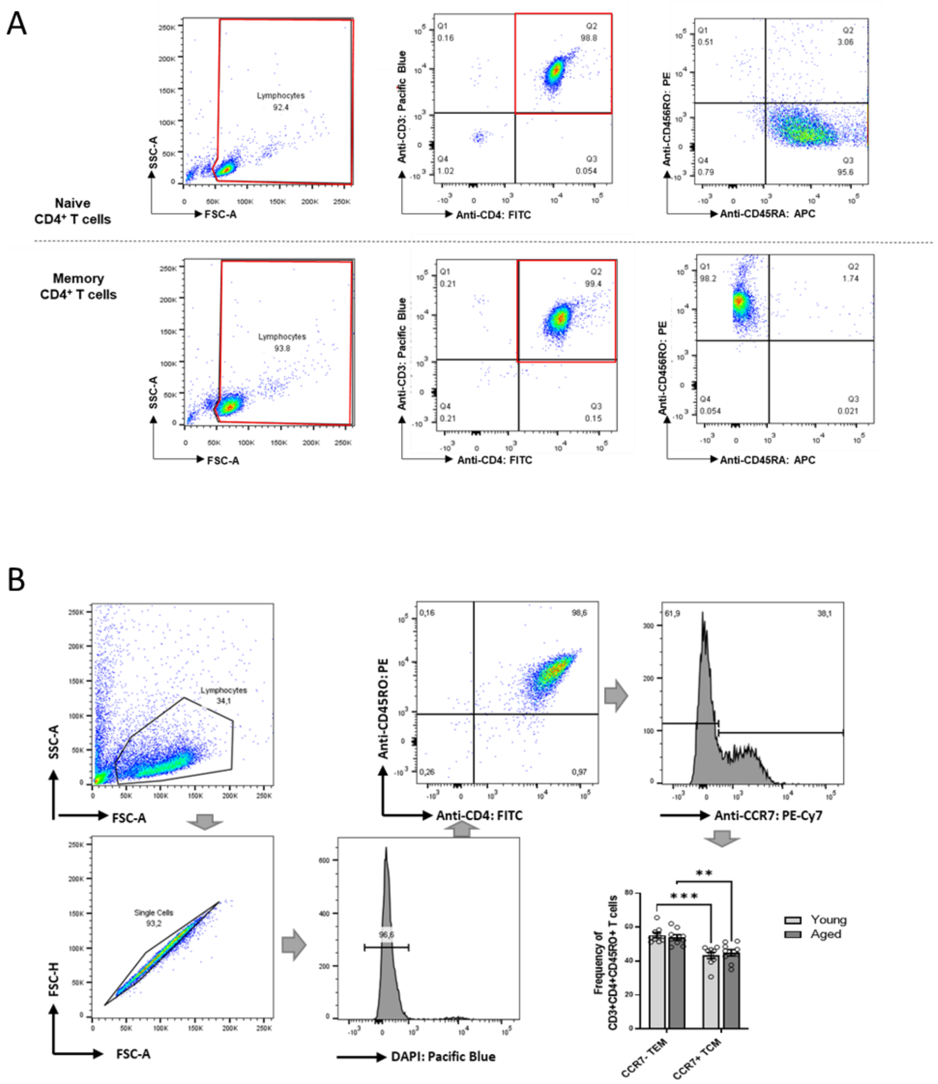

Supplementary Figure 1. Characterization and purity of naive and memory CD4⁺ T cells were verified by flow cytometry. (A) Representative example of the purity of naive and memory CD4⁺ T cells acquired by flow cytometry. Only naive and memory CD4⁺ T cells with a purity of > 95% were used in the experiments presented in this study. **(B)** Representative example CCR7 surface staining using flow cytometry (including gating) of freshly purified memory CD4⁺ T cells and comparison of the frequencies of CCR7⁻ effector memory CD4⁺ T cells and CCR7⁺ central memory CD4⁺ T cells (mean ± SEM; n = 9; 6 male and 3 female per group).

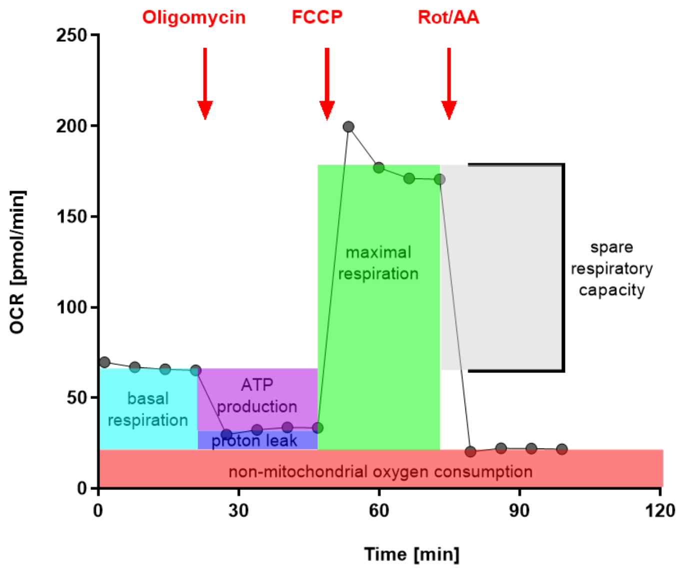

Parameter value	Equation	Interpretation
non-mitochondrial oxygen consumption	minimum rate measurement after Rotenone/antimycin A injection	Oxygen consumption persists due to a subset of cellular enzymes that continue to consume oxygen independent of mitochondrial respiration
basal respiration	(last rate measurement **before the first injection**) – (non-mitochondrial oxygen consumption)	Shows energetic demand of the cell under baseline conditions
proton leak	(minimum rate measurement after Oligomycin injection) – (non-mitochondrial oxygen consumption)	Proton leak can indicate mitochondrial damage or be used as a mechanism to regulate mitochondrial ATP production.
ATP production	(last rate measurement **before Oligomycin** injection) – (minimum rate measurement **after Oligomycin** injection)	Shows ATP produced by the mitochondria that contribute to meeting the energy needs of the cell
maximal respiration	(maximum rate measurement **after FCCP injection**) – (non-mitochondrial oxygen consumption)	Shows the maximum rate of respiration that the cell can achieve.
spare respiratory capacity	(maximal respiration) - (basal respiration)	The cell's ability to respond to demand can indicate cell fitness or flexibility, the capability of the cell to respond to an energetic demand as well as how closely the cell is to respire to its theoretical maximum
spare respiratory capacity as a %	(maximal respiration) / (basal respiration) × 100	above as a %
coupling efficiency	(ATP production) / (basal respiration) × 100	Efficiency to couple the oxidation of substrates to the phosphorylation of ADP to ATP.

Supplementary Figure 2. Seahorse XF Cell Mito Stress Test assay kinetic profile, parameters, parameter equation, and their interpretation. Taken from https://www.agilent.com/cs/library/usermanuals/public/Report_Generator_User_Guide_Seahorse_XF_Cell_Mito_Stress_Test_Single_File.pdf

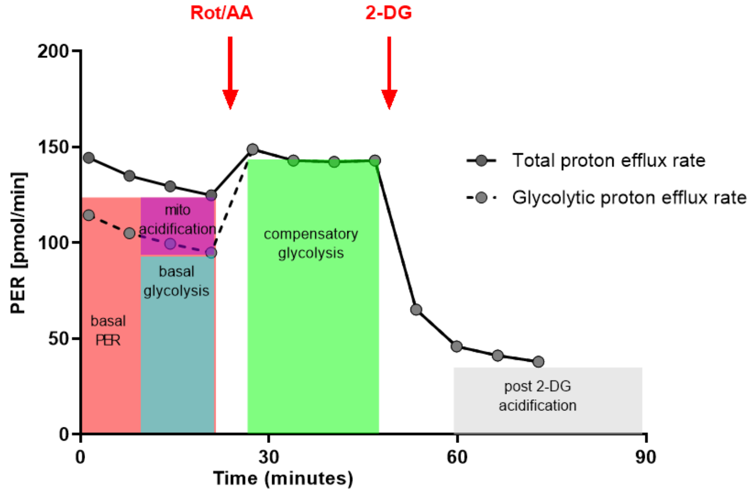

Parameter value	Equation	Interpretation
proton efflux rate (PER)	PER (pmol H+/min) = ECAR* (mpH/min) × BF* (mmol/l/pH) × geoVol* (µl) × Kvol*	delivers a quantitative measure of extracellular acidification
basal glycolysis	last glycoPER measurement **before the first injection**.	Proton Efflux rate derived from glycolysis discounting the effect of CO2-dependent acidification
basal PER	last PER measurement **before first injection**	Sum of mitochondrial CO2 - derived acidification and glycolytic contribution to PER
% PER from Glycolysis (Basal)	(basal glycolysis)/(basal PER) x 100%	calculates percent of acidification coming from glycolysis
Compensatory Glycolysis	maximum glycoPER measurement **after Rotenone/antimycin A (Rot/AA) injection**	The rate of glycolysis in cells following the addition of mitochondrial inhibitors, effectively inhibiting oxidative phosphorylation and driving compensatory changes in the cell to use glycolysis to meet the cells' energy demands.
mitoOCR/glycoPER (Basal)	[(last OCR measurement **before the first injection**) - (minimum OCR **after Rot/AA injection**)] /(basal glycolysis)	Ratio of oxygen consumption and
Post-2-DG acidification	Minimum glycoPER measurement after 2-DG injection	This value includes other sources of extracellular acidification that are not attributed to glycolysis or mitochondrial TCA activity as well as any residual glycolysis not fully inhibited by 2-DG. It is measured after the addition of 2-DG in the Glycolytic Rate Assay workflow.

* ECAR: Extracellular Acidification Rate [mpH/min]: rate of change in mpH in an assay well; BF: Buffer Factor [mmol/l/pH]: accounts for buffer capacity of both medium and sensor system; geoVol: geometric Volume [µl]: volume of the measurement microchamber assuming its completely sealed; Kvol: Volume scaling factor (no units): factor used to account for total proton production in the measurement chamber.

Supplementary Figure 3. Seahorse XF Glycolytic Rate assay kinetic profile, parameters, parameter equation, and their interpretation. Taken from https://www.agilent.com/cs/library/usermanuals/public/Report_Generator_User_Guide_Seahorse_XF_Glycolytic_Rate_Assay_SingleFile.pdf

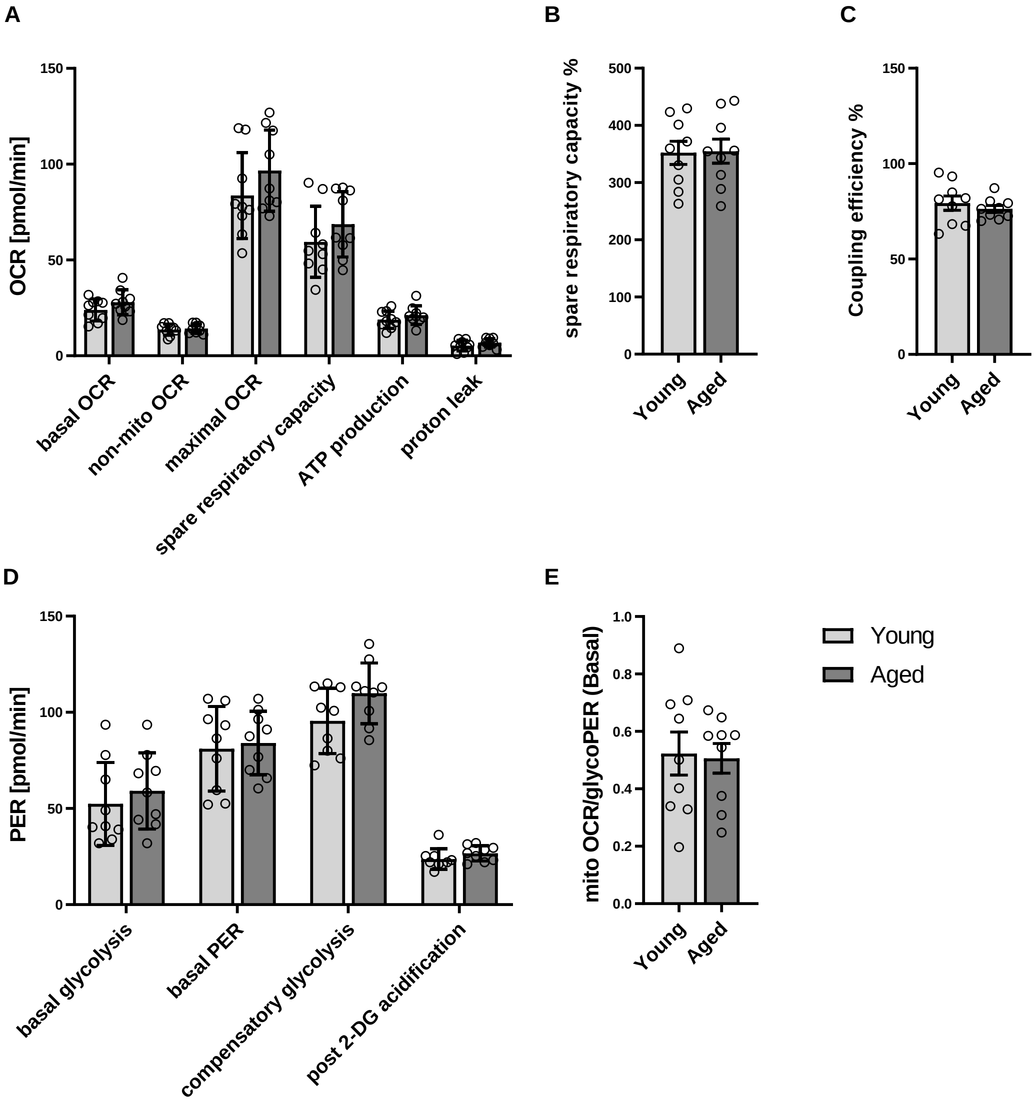

Supplementary Figure 4. Naive CD4+ T cells from young and aged donors present a similar mitochondrial metabolic and glycolytic phenotype. (**A**) Comparison of basal OCR, non-mito(chondrial) respiration, maximal respiration, spare respiratory capacity, ATP production, proton leak, (**B**) spare respiratory capacity%, and (**C**) coupling efficiency in naive CD4+ T cells from young donors and aged donors. (**D**) Comparison of basal glycolysis, basal PER, compensatory glycolysis, post-2-DG acidification, and (**E**) basal mitoOCR/glycoPER ratio in naive CD4+ T cells from young and aged donors. N=9 individual donors in each group. Data are shown as mean ± SD. An unpaired t-test was performed after the normality test. OCR: oxygen consumption rate; PER: proton efflux rate.

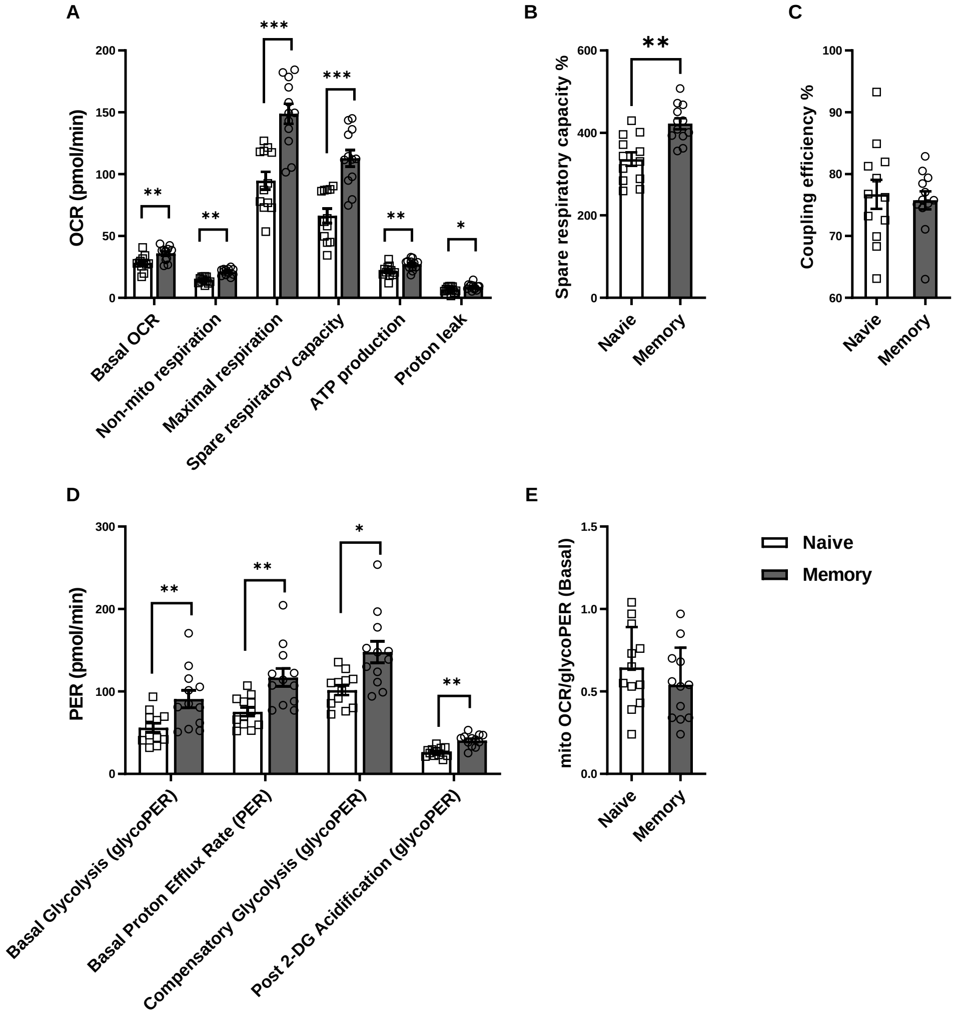

Supplementary Figure 5. Differences in the mitochondrial and glycolytic profiles between naive and memory CD4+ T cells in male donors. (**A**) Comparison of basal OCR, non-mito(chondrial) respiration, maximal respiration, spare respiratory capacity, ATP production, proton leak, (**B**) spare respiratory capacity%, and (**C**) coupling efficiency in naive and memory CD4+ T cells. (**D**) Comparison of basal glycolysis, basal PER, compensatory glycolysis, post 2-DG acidification rate, and (**E**) basal mitoOCR/glycoPER ratio in naive and memory CD4+ T cells (D, E). N=12 individual donors in each group. Data are shown as mean ± SD. A paired t-test was performed after the normality test. *P<0.05; **P<0.01; ***P<0.001. OCR: oxygen consumption rate; PER: proton efflux rate.

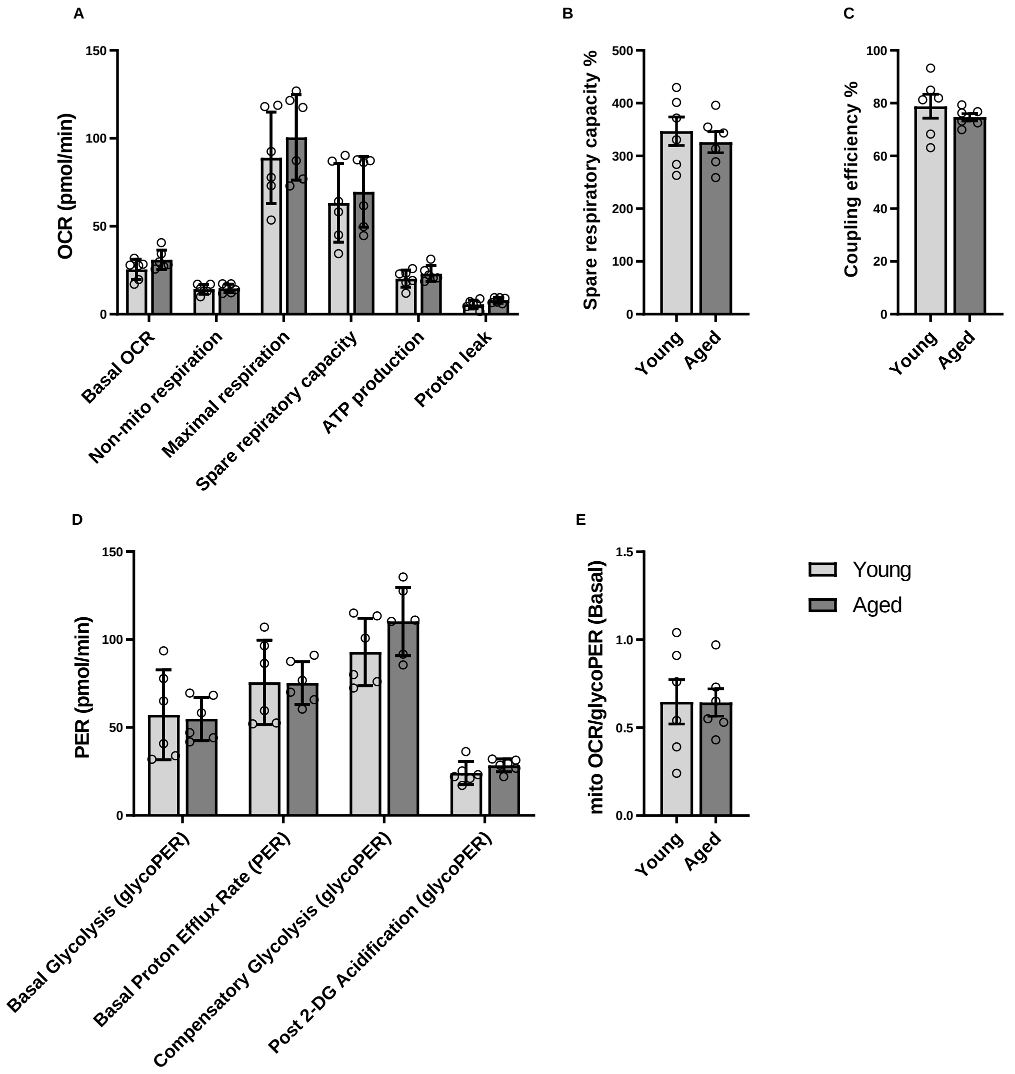

Supplementary Figure 6. Naive CD4+ T cells from young and aged male donors present a similar mitochondrial metabolic and glycolytic phenotype. (**A**) Comparison of basal OCR, non-mito(chondrial) respiration, maximal respiration, spare respiratory capacity, ATP production, proton leak, (**B**) spare respiratory capacity%, and (**C**) coupling efficiency in naive CD4+ T cells from young donors and aged donors. (**D**) Comparison of basal glycolysis, basal PER, compensatory glycolysis, post-2-DG acidification, and (**E**) basal mitoOCR/glycoPER ratio in naive CD4+ T cells from young and aged donors. N=6 individual donors in each group. Data are shown as mean ± SD. An unpaired t-test or unpaired t-test with Welch’s correction was performed after the normality test. OCR: oxygen consumption rate; PER: proton efflux rate.

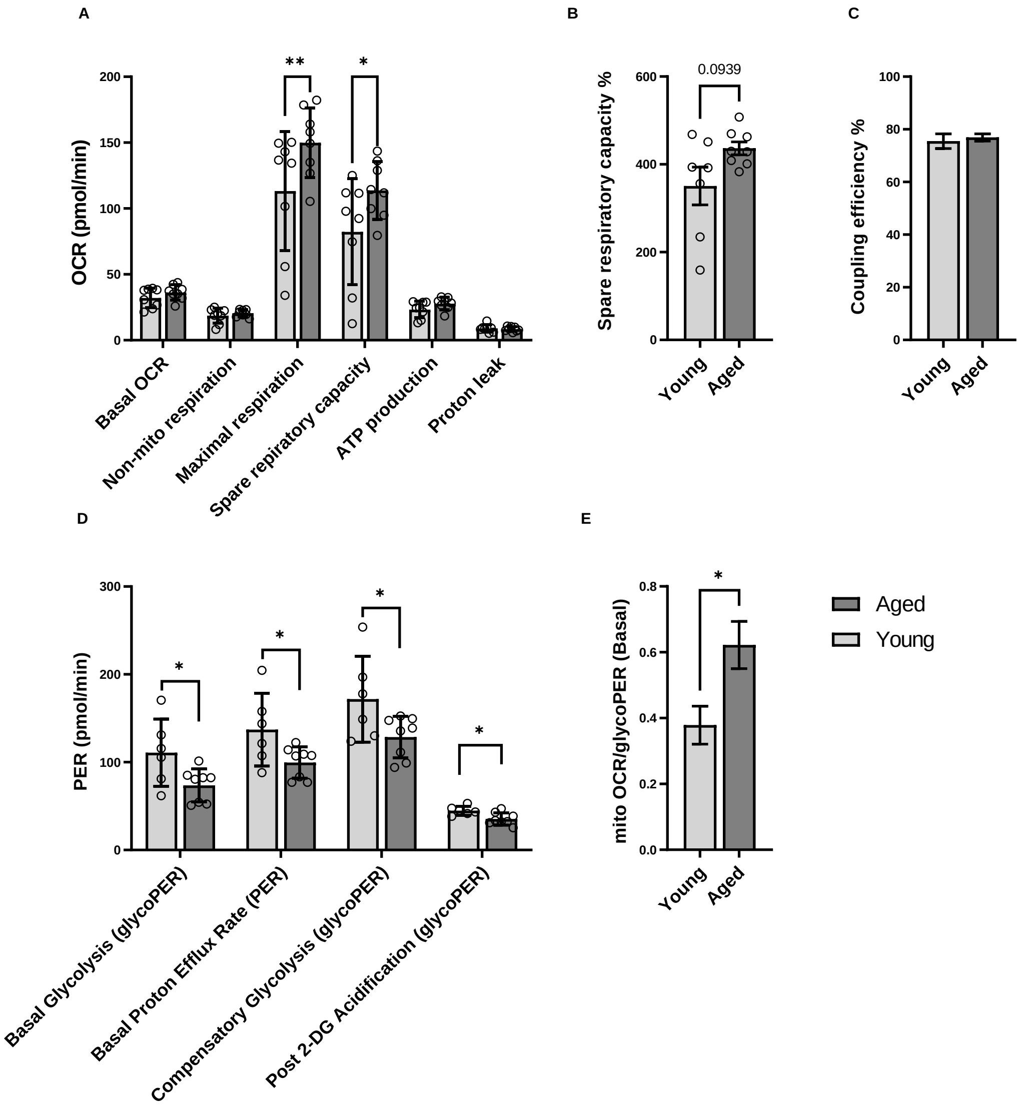

Supplementary Figure 7. Differences in the mitochondrial and glycolytic profile of memory CD4+ T cells between young and aged male donors. (**A**) Comparison of basal OCR, non-mito(chondrial) respiration, maximal respiration, spare respiratory capacity, ATP production, proton leak, (**B**) spare respiratory capacity%, and (**C**) coupling efficiency in naive CD4+ T cells from young and aged donors. (**D**) Comparison of basal glycolysis, basal PER, compensatory glycolysis, post-2-DG acidification, and (**E**) basal mitoOCR/glycoPER ratio in naive CD4+ T cells from young and aged donors. Young group: n=6 individual donors; aged group n=8 individual donors. Data are shown as mean ± SD. After the normality test, an unpaired t-test or Mann- Whitney test was performed. *P<0.05. OCR: oxygen consumption rate; PER: proton efflux rate.
